# Applications of large-scale molecular profiling techniques to the study of the corpus luteum

**DOI:** 10.21451/1984-3143-AR2018-0038

**Published:** 2018-08-03

**Authors:** Joy L. Pate, Camilla K. Hughes

**Affiliations:** Penn State University, Center for Reproductive Biology and Health, Department of Animal Science, University Park, PA 16802 USA

**Keywords:** bovine, corpus luteum, molecular profiling.

## Abstract

The corpus luteum (CL) is vital for the establishment and maintenance of pregnancy. Throughout the history of luteal biology, cutting-edge technologies have been used to develop a thorough understanding of the functions of specific luteal cell types, the signaling pathways that result in luteal cell stimulation or demise, and the molecules that regulate specific functions of luteal cells. The advent of large- scale profiling technologies such as transcriptomics, proteomics, and metabolomics, has brought with it an interest in discovering novel regulatory molecules that may provide targets for manipulation of luteal function or lifespan. Although the work to date is limited, transcriptomics have been effectively used to provide a global picture of changes in mRNA that relate to luteal development, steroidogenesis, luteolysis or luteal rescue. Some studies have been reported that profile microRNA (miRNA) and proteins, and although not yet published, metabolomics analyses of the CL have been undertaken. Thus far, these profiling studies seem to largely confirm earlier findings using targeted approaches, although previously unstudied molecules have also come to light as important luteal regulators. These molecules can then be studied using traditional mechanistic techniques. Use of profiling technologies has presented physiologists with unique challenges associated with analyses of big data sets. An appropriate technique for balancing the risks associated with type I (false discoveries) and type II (overlooking a real change) statistical error has not yet been developed and many big data studies may have potentially important differences that are overlooked. Also, it is imperative that attempts be made to integrate information from the various -omics studies before drawing conclusions based on expression of only one class of molecule, to better reflect the interdependency of molecular networks in cells. Currently, few analysis programs exist for such integrations. Despite challenges associated with these techniques, they have already provided new information about the biology of the CL, notably allowing identification of a key regulator of acquisition of luteolytic capacity and providing a big-picture view of the subtle changes that occur in the CL during early pregnancy. As these technologies become more accurate and less expensive, and as analysis becomes more user- friendly, their use will become much more widespread and many new discoveries will be made. This review will focus only on relevant studies in which these technologies were used to study the CL of ruminants.

## Introduction

In the 1600’s, Regnier de Graaf described his observation of transient yellow globules that form from emptied ovarian follicles after coitus, noting that the number of globules was the same as the number of fetuses (Jocelyn and Setchell, 1972), and Marcello Malpighi first called this structure a corpus luteum, latin for yellow body. The function of the corpus luteum (CL) remained a mystery for 300 years, when experimental evidence was obtained that the CL was necessary for the maintenance of pregnancy (Simmer 1971; Frobenius 1999). This was followed by the discovery of the primary secretory product of the CL, progesterone, in the 1930’s. Despite a slow beginning to the understanding of the function of the CL, once it was identified that this small structure was absolutely essential for the establishment and maintenance of pregnancy in all mammals, it captured the attention of reproductive biologists, and none more so than those interested in reproduction of domestic ruminants. Thus, in the last 50 years, great advances have been made in understanding luteal function, much of which came from studies in cows and sheep. While the goal of this research was to enhance fertility of these species, the knowledge of the basic biology of the CL could be generally applied to nonruminants, and because of its ephemeral nature, the CL has served as a model for many aspects of cellular biology, including angiogenesis, tumor development, steroidogenesis, roles of tissue-resident immune cells, and pathways of cellular death.

During the mid- to late 20th century, the hormonal regulators, second messenger molecules and biochemical reactions in steroidogenesis were elucidated. Sources of cholesterol as substrate for progesterone synthesis and intracellular signaling pathways were defined. Refined procedures to separate cells based on size led to a race to determine the origins and distinct functions of the small and large steroidogenic cells, and the discovery that oxytocin is produced in the CL prompted a flurry of research to determine if luteal oxytocin is necessary for uterine prostaglandin (PG)F2A release during luteolysis. The cellular heterogeneity that characterizes the CL also intrigued researchers, whose work revealed the contributions of endothelial cells, fibroblasts, pericytes and immune cells to development, function, and regression of the CL. The advent of technologies for identifying and quantifying steady state concentrations of mRNA in cells and tissue, including northern blotting, PCR, and qPCR, brought about a revolution in targeted-approach experimentation to elucidate how changes in luteal functions are driven by changes in gene expression. For more information about these discoveries and the general biology of the CL, the reader is referred to a number of reviews on ruminant luteal function (Niswender *et al*., 2000; Pate *et al*., 2012; Wiltbank *et al*., 2012; Miyamoto *et al*., 2013; Smith and Meidan, 2014).

## Transcriptomic profiling in the corpus luteum

Using the technologies mentioned above, studies of mRNA concentrations in the CL have been hypothesis-driven, searching for the key changes in mRNA relative to receptor activation, signal transduction, steroidogenesis, cytokine production, and cell death pathways. Much has been learned about which pathways and genes were regulated during development and regression of the CL using this type of approach. However, more recently, researchers have used high throughput technologies to profile many (microarray) or all (sequencing) of the transcripts present in the CL from selected times or physiological states. This approach was at first criticized as being a fishing expedition, but identification of potentially important molecules that led to new hypotheses about luteal regulation has enhanced acceptance of these powerful approaches. Transcriptomic analyses have largely confirmed our understanding of luteal functions as determined by more targeted approaches, lending further support to previously drawn conclusions. Perhaps more importantly, they have also shed light on unexplored or potentially new cellular pathways and functions.

Development of the ruminant CL involves differentiation of follicular steroidogenic cells and it was suggested from cell-labeling studies that the small luteal steroidogenic cells (SLC) are derived from the thecal cells of the follicle, whereas the large cells (LLC) originally differentiate from granulosal cells (Alila and Hansel, 1984). Romereim *et al*. (2017) used microarrays to profile the transcriptomes of isolated granulosal, thecal, and separated luteal cells. This approach supported the existing model of differences between SLC and LLC, including identification of the LHCGR in greater abundance on SLC and the PTGFR in greater abundance on LLC. Additionally, it allowed for identification of six novel cell lineage markers each for the thecal cell-SLC lineage and the granulosal cell-LLC lineage. These lineage markers include molecules involved in ion and molecular transport and lysosomal function in LLC and are primarily molecules involved in signaling in SLC. Further, the transcriptome of the large steroidogenic cells indicated that these cells likely function in recruitment of immune and endothelial cells, activities that had not previously been ascribed to a particular luteal steroidogenic cell type. Baddela *et al*. (2018) reported 1276 differentially abundant mRNA in small and large luteal cells. The small luteal cells were enriched in mRNA responsible for immune cell recruitment, whereas the profile of large luteal cell mRNA suggested functions in regulating folliculogenesis, luteolysis, and small molecule metabolism. The reported purity of the separated cell populations was similar in these two studies, so the clear discrepancy between them may be due to the cell-type comparisons made. Baddela *et al*. used days 11-12 CL (n = 4) from timed estrous cycles. The stage of the cycle from which CL (n = 3) were collected in the study of Romereim *et al*. was not described. Because differentiation of the small and large cells is a somewhat continuous process, it is possible that functions associated with small and large cells are stage- dependent. Although it remains to be determined which steroidogenic cell type is responsible for recruiting immune cells, other differentially abundant mRNA and predicted functions of small and large cells were fairly consistent between the two studies.

Differentiation and maximal steroidogenic capacity of the ruminant CL is dependent on luteinizing hormone. As might be expected, gonadotropic stimulation of the CL resulted in upregulation of genes related to lipid metabolism, cholesterol metabolism and progesterone production (Fatima *et al*., 2012). The most upregulated mRNA was fatty acid binding protein 5 (FABP5), which can transport lipids within cells to lipid droplets and mitochondria. To our knowledge, this potentially important regulator of steroidogenesis has not been studied in the CL. Transcriptomic analysis of day 4 and day 11 bovine CL also indicated that steroidogenic and cholesterol biosynthetic genes are upregulated in the midcycle CL, along with genes involved in immune response, whereas the day 4 CL is characterized by genes related to cell cycle, DNA replication and metabolic processes (Kfir *et al*., 2018). This analysis also revealed that the developing CL expresses angiogenesis-promoting genes, whereas the mature CL expressed genes related to cessation of blood vessel sprouting.

As the CL develops, it must gain the capacity to regress in response to PGF2A (Tsai and Wiltbank, 1998). The inability of the developing CL to regress, despite clear responses to PGF2A, has intrigued ovarian biologists for decades, leading to comparison of the transcriptomes of early (day 4) and midcycle (dday 10) CL in response to exogenous PGF2A. Using microarrays, Goravanahally *et al*. (2009) found 167 differentially expressed genes, most of which were upregulated in the day 10 CL, likely reflecting differentiation and development of pathways for maximal steroidogenesis. This group then focused on the 20 genes that were associated with cell signaling pathways, as these genes could be regulators of luteal responsiveness to PGF2A. Collection of CL 24 h after a luteolytic injection of PGF2A showed upregulation of CAMKK2 in day 9, but not day 4, CL. Although the CL in this study were collected 24 h after the PGF2A injection, when luteolysis is advanced, this research group later showed that CAMKK2 is indeed a component of the PGF2A signaling pathway in day 10, but not day 4, CL (Bowdridge *et al*., 2015), providing a good example of how a profiling experiment led to the discovery of a molecule that could potentially be targeted to regulate luteal function. Differences in PGF2A regulation of gene transcription were further delineated by Mondal *et al* (2011) who also used microarrays to determine differentially abundant genes in early (day 4) and midcycle (day 11) CL collected following administration of PGF2A. Prostaglandin- regulated genes were detected in both types of CL, but the response was much more robust in the midcycle CL. Genes that were upregulated by PGF2A in day 11, but not day 4, CL indicated activation of biological processes involved in receptor activity, cellular death, and immune cell-related genes, and many were genes that are under the control of ETS family transcription factors. This is a large family of transcription factors that is associated with regulation of many cellular functions, including apoptosis. Of note, almost all of the listed biological processes upregulated by PGF2A within 4 h in the midcycle, but not day 4 CL, include genes normally associated with an immune response, such as cytokines, chemokines and adhesion molecules. A macrophage marker, CD14, was upregulated by PGF2A on day 11, but not day 4, suggesting differential recruitment of immune cells into the mature CL, after induction of luteal regression. The profiling study of Mondal *et al*. (2011) also led to further studies that defined the response of angiogenic factors to PGF2A in day 4 and day 11 CL and their functional roles on luteal endothelial cells (Zalman *et al*., 2012). Overall, these studies support the earlier work from Wiltbank’s group (Tsai and Wiltbank, 1998) showing that the day 4 CL is not unresponsive to PGF2A, rather the responses of the day 4 CL differ from those of an older CL that has acquired luteolytic capacity.

Shah *et al*. (2014) and Talbott *et al*. (2017) used microarrays to investigate temporal changes in gene expression during luteal regression, using water buffalo and cows, respectively. As in previous studies, changes in transcripts related to steroidogenesis, LH receptor signaling, and apoptosis were observed. Talbott *et al*. (2017) also confirmed earlier studies in which progesterone declined prior to any decrease in transcripts related to steroidogenesis, and in which luteolysis was characterized by changes in transcripts related to cholesterol availability. However, most of these transcripts also changed coincident to, not before, the decrease in progesterone. Transcription factor mRNA and transcripts indicating activation of cytokine signaling were altered prior to the decrease in progesterone, further supporting the growing evidence that inflammatory-like events are key mediators of PGF2A-induced luteolysis (Mondal *et al*., 2011; Atli *et al*., 2012; Shah *et al*., 2014; Talbott *et al*., 2017). Shah *et al*. also reported downregulation of CYP19A1 and differential abundance of estrogen-responsive genes, a novel finding. Although estrogen synthesis by the bovine CL is low, a role of intraluteal estrogen and estrogen receptor signaling in PGF2A-induced luteolysis was proposed. This would extend previous findings that estrogen can induce premature luteolysis (Wiltbank *et al*., 1961) and that follicular estradiol may be necessary for timing normal luteolysis (Villa-Godoy *et al*., 1985).

**Figure 1:**
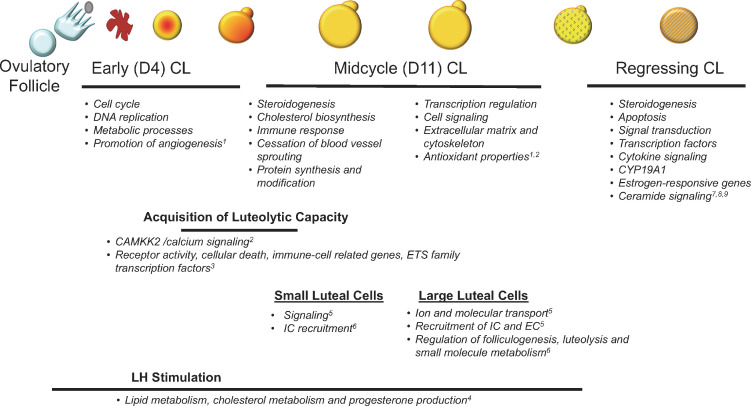
Pathways associated with stages of luteal development, maintenance and regression as revealed by transcriptomic profiling of ruminant CL. Superscripts refer to references as follows: ^1^Kfir *et al*., 2018; ^2^Goravanahally *et al*., 2009; ^3^Mondal *et al*., 2011; ^4^Fatima *et al*., 2012; ^5^Romereim *et al*., 2017; ^6^Baddela *et al*., 2018; ^7^Talbott *et al*., 2017; ^8^Shah *et al*., 2014; ^9^Ochoa *et al*., 2018.

Atli *et al*. (2012) developed a model of repeated intrauterine infusions of physiological concentrations of PGF2A, coupled with luteal biopsies, to evaluate temporal changes in the CL during luteal regression. This study, which used qPCR to profile transcripts, indicated that activation of genes related to immune response and prostaglandin metabolism were necessary to ensure the progression of luteolysis. This model was further used to determine if PGE2, which is thought to be involved in luteal rescue during early pregnancy, could suppress PGF2A-induced gene expression. The magnitude of effect of PGF2A pulses on gene expression in the CL, as assessed using RNAseq, was quite large (Ochoa *et al*., 2018). Compared to saline infused controls, 572 mRNA were altered by PGF2A, with an additional 373 mRNA that differed from PGE2 and PGF2A + PGE2 infusions. Transcripts most significantly regulated by PGF2A included those associated with steroidogenesis, apoptosis, and signal transduction, as expected. These data also indicated that ceramide signaling may be associated with luteolysis. Remarkably, compared to saline controls, there were no differentially abundant mRNA in the CL following intrauterine infusions of PGE2 or PGE2 + PGF2A, and these CL did not regress, demonstrating that PGE2 can completely prevent PGF2A-induced changes in mRNA that would ensure luteolysis. A summary of the pathways associated with the stages of luteal development, maintenance and regression, as determined by RNA profiling of luteal tissue, is depicted in Fig. 1.

Little is known about changes that occur within the CL to facilitate its rescue during early pregnancy. When the CL of pregnancy was compared to midcycle (days 10-12) CL, differentially abundant mRNA gradually increased throughout pregnancy (Sakumoto *et al*., 2015), indicating that, once rescued, the CL is not static, but is actively regulated by either intrinsic or extrinsic factors that alter mRNA abundance to facilitate luteal survival and progesterone production. In this study, large changes in chemokine mRNA in the CL of pregnancy were noted, particularly a more- than 10-fold decrease in lymphotactin, a chemokine that recruits T cells, and a more-than 100-fold increase in eotaxin, a chemokine that recruits eosinophils. This study also noted a more modest increase in growth factor-related mRNA during early pregnancy (Sakumoto *et al*., 2015). Similarly, a microarray study performed by Romero *et al*. (2013) demonstrated that in the ovine CL of early pregnancy, there is stabilization or upregulation of pathways related to interferon and cytokine signaling, cell-cell adhesion, and cytoskeleton, as compared to the late and regressing CL. Pentraxin-3, which is produced by several immune cell types, was stabilized in early pregnancy, but reduced during luteal regression. The authors suggest that this molecule may increase cellular resistance to stress (Romero *et al*., 2013). Overall, these and other studies demonstrate that chemokines and cytokines appear to be key regulators of both luteal regression and luteal survival during pregnancy.

The advent of RNAseq provided more sensitive and more accurate detection of mRNA, allowing for more comprehensive studies of the luteal transcriptome, and providing the opportunity to reveal potentially important transcripts that were previously unrecognized. In a recent RNAseq study comparing transcript abundance in bovine CL of day 17 of the estrous cycle and day 17 of pregnancy, 144 differentially abundant mRNA were reported and immune signaling pathways were among those predicted to be modulated in early pregnancy, as well as novel potential regulators of luteal rescue, including PPAR signaling and PDGF signaling (Hughes *et al*., 2018; Penn State University, Center for Reproductive Biology and Health, University Park, PA USA; unpublished data) Moore *et al*. (2016) used RNAseq to determine if CL and endometria of cattle of low or high genetic merit for fertility contained differentially abundant mRNA. Only 9 mRNA were different in the endometrium, whereas 560 mRNA were different in the CL, suggesting an important relationship between luteal function and fertility. Of the 560 DE mRNA, 85% were lesser in abundance in the CL from low fertility cows, indicating a general reduction in luteal activity. These included genes related to steriodogenesis, extracellular matrix and RNA replication, indicating compromised luteal development and steroidogenic capacity. Conversely, mRNA related to PGF2A response were greater in CL from low fertility cows.

**Figure 2:**
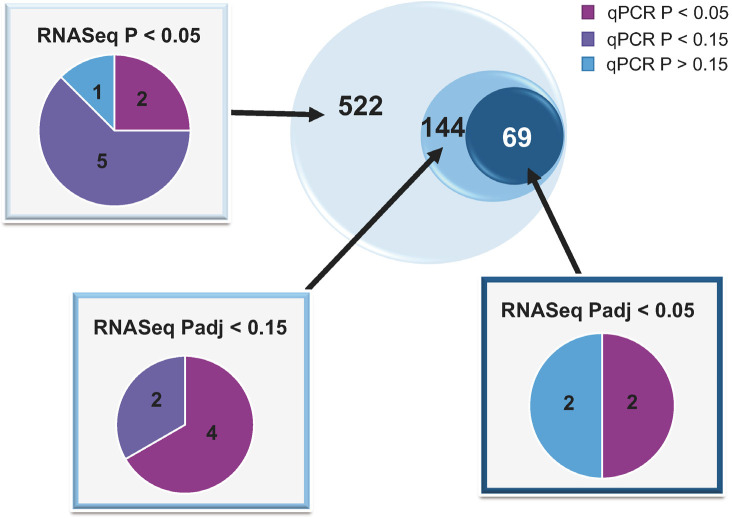
mRNA that were differentially abundant in a transcriptomics study (Hughes *et al*., 2018; Penn State University, Center for Reproductive Biology and Health, University Park, PA USA; unpublished data). Three P-value cutoffs were used (P < 0.05, padj < 0.15, padj < 0.05), with 522, 144, and 69 mRNA in each group. Padj-values are P-values that have been adjusted for false discovery rate of 5% false discoveries. These three groups are represented by the three concentric circles. A subset of mRNA from each group was analyzed using qPCR (n = 6); total number of mRNA analyzed by qPCR in each subset is represented within each pie chart as significantly (P < 0.05), or with a tendency to be (P < 0.15), differentially expressed, or not DE (P > 0.15).

Although a primary focus in statistical analysis of transcriptomic datasets has been on the reduction of type I error, due to the large number of statistical tests performed, this problem has been largely corrected by the Benjamini Hochberg false discovery rate correction, which allows a researcher to choose a threshold (typically between 5 and 15%) of false discoveries that they are willing to tolerate (Benjamini and Hochberg, 1995). However, while this method reduces type I error, it increases type II error and, in some studies, may cause researchers to overlook many genes, proteins, or metabolites that change in a biologically relevant way (Mudge *et al*., 2017). Recent experiments have demonstrated that in an RNAseq study of the CL of the estrous cycle and pregnancy, mRNA that were well below the false discovery rate cutoff were still differentially expressed (P < 0.05) by two-fold or more when measured by qPCR (Hughes *et al*., 2018; Penn State University, Center for Reproductive Biology and Health, University Park, PA USA; unpublished data; Fig. 2). Given the cost and time associated with gene expression profiling experiments, the amount of information lost due to this increased type II error is concerning. One proposed solution to this problem is an optimized P- value cutoff, based on power and relative cost of type I and type II error, for each big data study (Mudge *et al*., 2017).

## MicroRNA profiling and regulation of the corpus luteum

MicroRNA are single-stranded noncoding RNA, approximately 22 nt in length, that serve as posttranscriptional regulators of gene expression. Following transcription, the precursor forms of miRNA undergo nuclear and cytoplasmic cleavage to form the mature miRNA. The mature miRNA binds to Argonaute 2 (AGO2), is then incorporated into the RNA-Induced silencing Complex (RISC), and the complex is targeted to the 3’UTR of mRNA with sufficient complementarity to the miRNA. This results in loss of mRNA translation to protein, either via translational repression or degradation of the mRNA. Individual miRNA can have numerous mRNA targets, and individual mRNA can be targeted by many miRNA, which makes elucidation of miRNA- regulated signaling pathways quite complex. MicroRNA expression and activity can be both tissue- and stage- specific, and in some cases can even enhance, rather than suppress, synthesis of specific proteins. The complexity of miRNA biology necessitates confirmation of a proposed regulatory role in a particular tissue at a specific developmental or functional stage. For information on microRNA biology, the reader is referred to reviews by He and Hannon (2004), Treiber *et al*. (2012), Catalanotto *et al*. (2016), Maalouf *et al*. (2016a), and Tesfaye *et al*. (2018).

MicroRNA have captured the attention of biologists in many disciplines, but in particular, seem to have improved the understanding of the dynamic nature of reproductive tissues. Most studies of ovarian miRNA have focused on follicles and oocytes of nonruminant species (reviews: Christenson, 2010; Hossain *et al*., 2012; Li *et al*., 2015; McGinnis *et al*., 2015; Maalouf *et al*., 2016a). Hossain *et al*. (2009) cloned and sequenced a small RNA library derived from bovine ovary and found that some miRNA were dissimilar in abundance among cortex, follicles and CL. Using a screened target set of 115 genes likely to be regulated by abundant ovarian miRNA, pathway analysis indicated that the selected miRNA and their predicted targets were indeed involved in functions indicative of the dynamic nature of ovarian components, such as growth factor signaling, cellular growth and development, and cellular death.

Expression of miRNA in the CL may be developmentally regulated, because greater abundance of miRNA in the mature than in the developing CL has been reported (Maalouf *et al*., 2016b; Baddela *et al*., 2017), and the functions regulated by luteal miRNA shift from cellular metabolism and growth in the day 4 CL to cell cycle, cell death, and gene expression in the midcycle CL (Maalouf *et al*, 2016b). One of the upregulated miRNA, miR34a, targeted NOTCH1 and YY1 and promoted luteal cell progesterone production while suppressing proliferation of luteal cells (Maalouf *et al*., 2016b), consistent with a role in inhibition of growth while enhancing differentiated function. McBride *et al*. (2012) reported that 9 miRNA decreased and 8 increased during the follicular-luteal transition in sheep, and their predicted targets are involved in cellular development, differentiation, proliferation and survival. The difference in number and direction of DE miRNA between these two studies is likely due to method of detection (microarray *vs*. Sanger sequencing) and comparison of mature CL to follicular (McBride *et al*., 2012) *vs*. immature luteal (Maalouf *et al*., 2016b) cells. When water buffalo CL of 3 estrous cycle stages were compared to granulosal cells using miRNAseq, more miRNA were CL-specific than granulosal cell- specific and 39 of 43 differentially abundant miRNA were greater in abundance in CL. Interestingly, 93% of the luteal-unique miRNA mapped to a 0.7 Mb region of buffalo chromosome 20 (chromosome 21 of cows) and it was proposed that this miRNA cluster suppresses 20- alpha hydroxysteroid dehydrogenase, and thus progesterone metabolism, during luteinization (Baddela *et al*., 2017). In one study, a greater abundance of miRNA was found in follicular cells compared to luteal cells (Mohammed *et al*., 2017). Perhaps there is a robust expression of miRNA in developing and preovulatory follicles, that is then generally downregulated around the time of ovulation and early luteinization. Subsequent upregulation of miRNA during latter developmental stages of the CL would negatively regulate growth and support maximal steroidogenesis. It should be noted that in one recent study, there was no change in the number of miRNA in the midcycle compared to the early CL (Gecaj *et al*., 2017). It is unclear why this study differs from the previous ones.

Ma *et al*. (2011) compared nonregressed to regressed CL and reported 13 DE miRNA, 7 being less abundant and 6 being more abundant in regressed CL. The most downregulated miRNA in regressed CL was miR378, and its expression appeared to be inversely correlated to its predicted target, IFNGR1 protein, suggesting that it may serve to repress IFNG-mediated cell death in the nonregressed CL. Using next generation sequencing, Maalouf *et al*. (2014) identified 544 known and 46 novel miRNA in the bovine CL. To determine if miRNA may be involved in luteal rescue during maternal recognition of pregnancy, CL collected on day 17 of the estrous cycle were compared to CL collected on the same day of pregnancy. Fifteen miRNA were found to be differentially abundant. The predicted targets of these 15 miRNA represent genes involved in immune-related events and apoptosis, reminiscent of pathways predicted to regulate luteal survival in the transcriptomic studies mentioned above. One of the miRNA targets associated with the top pathways in this study was CAMKK1, which, along with the mediator of acquisition of luteolytic capacity discussed previously, CAMKK2, plays a role in the calcium/calmodulin- dependent (CaM) kinase cascade. This study indicated that miRNA are also likely to play in role in luteal rescue (Maalouf *et al*., 2014).

A comprehensive study of miRNA expression using miRNAseq has shown that some miRNA are highly abundant throughout luteal lifespan, whereas others are found in abundance only at specific stages (Gecaj *et al*., 2017). The dynamic and transitory nature of the CL makes it an exemplary tissue for demonstration of stage specificity of miRNA expression. The top 20 most abundant miRNA (based on mean reads from all stages of the cycle studied in each study) were identified for four miRNA profiling studies (Maalouf et al., 2014, 2016b, Baddela et al., 2017, Gecaj et al., 2017). MiRNA found in only one study, or common to two, three, or all four studies are listed in Table 1. The five miRNA common to at least three of these studies were analyzed in mirPath version 3 (Vlachos et al., 2015) and the top 10 gene ontology (GO) terms associated (P < 10^-325) with their predicted targets, using the Tarbase database, are listed in Table 2. The top 10 GO terms indicate that the most abundant miRNA in the CL are likely to be involved in regulation of the cell cycle, protein synthesis, and immune function. Notably, all five miRNA have predicted targets associated with each of the top 10 GO terms, demonstrating possible redundancy in miRNA functions.

Finding significant differential expression of miRNA using profiling techniques may be affected by sample size, statistical analyses, isomiR distribution, relative abundance, and degree of variation among biological replicates. Thus, it is not surprising when some discrepancies in lists of DE miRNA in different studies occur. Researchers must use caution when drawing definitive biological conclusions based on somewhat arbitrary cutoffs for significance and variation in how data are handled.

Although profiling technologies have significantly enhanced understanding of miRNA in the corpus luteum, the targeted approaches that have sprung from these miRNA profiling studies have yielded important functional information about specific miRNA in the CL. Dai *et al*. (2014) reported an increase in the abundance of miR126 during luteal development, during which time its expression was inversely correlated to Talin2, suggesting that miR126 may regulate cellular interactions with extracellular matrix during final maturation of the CL. Maalouf *et al*. (2016b) also observed greater abundance of miR126 in midcycle compared to developing CL. miR96 is upregulated in the early CL compared to the follicle and supports survival of luteal cells by directly targeting FOXO1 (Mohammed *et al*., 2017). Angiogenesis in the developing CL is at least partially regulated by miR221 targeting thrombospondin 1 in luteal endothelial cells (Farberov and Meidan, 2017).

**Table 1:** miRNA identified among the top 20 most abundant miRNA in at least one of four miRNA profiling studies.

Number of studies	miRNA
Four	let-7a-5p
Three	mir-21-5p, let-7f, mir-26a, let-7b
Two	let-7c, let-7d, let-7e, let-7g, let-7i, mir-100, mir-103, mir-10b, mir-125b, mir-143, mir-148a, mir-202, mir-30d, mir-320a, mir-3600
One	let-7j, mir-107, mir-126-3p, mir-126-5p, mir-127, mir-140, mir-145. mir-148b, mir- 151-3p, mir-154c, mir-1839, mir-186, mir-199a-3p, mir-214, mir-2284x, mir-23b, mir-24a, mir-26c, mir-27b, mir-29a, mir-30a-5p, mir-30e-5p, mir-320b, mir-320c, mir-342, mir-378, mir-423-5p, mir-450a, mir-486, mir-503-5p, mir-99a-5p, mir-99b

**Table 2:** Top 10 gene ontology (GO) terms associated with predicted targets of the 5 miRNA common to at least 3 of the studies.

GO Category	Number of predicted target genes associated with GO category
G1/S transition of mitotic cell cycle	69
G2/M transition of mitotic cell cycle	64
Mitotic cell cycle	188
Protein binding transcription factor activity	199
Nucleic acid binding transcription factor activity	292
Toll-like receptor signaling pathway	51
Immune system process	430
MyD88-independent toll-like receptor signaling pathway	42
Molecular function	4339
RNA binding	702

## Proteomic profiling in the corpus luteum

**Figure 3:**
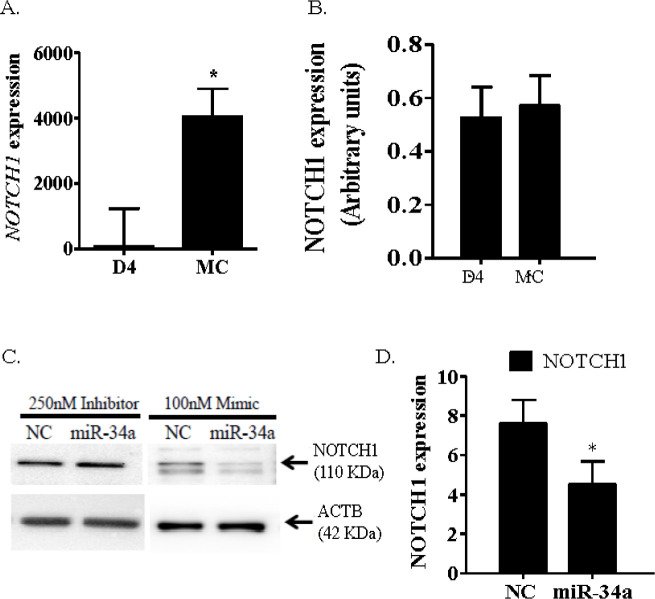
Relative expression of *NOTCH1* (mRNA, A) and NOTCH1 (protein, B) in developing (day 4) and fully functional (MC=midcycle, days 10-12) CL. C) Representative western blot depicting downregulation of NOTCH1 in response to a miR-34a mimic compared to a negative control (NC) scrambled sequence RNA, and D) Mean (n = 3) NOTCH1 protein abundance in response to miR-34a mimic. Adapted with permission from Maalouf *et al*. (2016b).

Inverse correlation of miRNA and mRNA abundance is often used as evidence that a particular mRNA is a target of a miRNA of interest. However, it is not always the case that a miRNA-mRNA interaction results in degradation of the target mRNA. If instead, the interaction simply causes translational repression of the mRNA, the steady state concentration of the mRNA may remain unchanged, or may increase if expression of the gene continues. Therefore, confirmation of a miRNA target requires determination of a change in protein concentration when the miRNA concentration is altered. An example of this is NOTCH1 regulation by miR34a in the CL (Fig. 3). Both miR34a and *NOTCH1* mRNA are greater in the midcycle than the developing CL, but the increase in *NOTCH1* is not reflected by an increase in NOTCH1 protein, indicating either translational repression or enhanced protein degradation. In cultured luteal cells, a miR34a mimic clearly decreased NOTCH1, confirming translational repression by this miRNA. Therefore, global investigation of miRNA targets will be more reliable using proteomic, rather than transcriptomic, analyses.

Few proteomics studies of the CL have been conducted and proteomic studies present challenges in terms of sensitivity. Often, identified proteins represent only the most highly abundant fraction of total proteins in a tissue. The two published studies of proteomics in the ruminant CL used two-dimensional polyacrylamide gel electrophoresis and MALDI-TOF-MS to identify proteins that changed during the estrous cycle or pregnancy. Arianmanesh *et al*. (2011) found that 139 proteins were upregulated and 69 were downregulated in the ovine CL as it progressed from day 12 to day 16 of the cycle. On day 16, plasma progesterone was low, indicating that the CL was regressed, but upregulated proteins included those involved in signal transduction, oxidative stress and structural integrity, indicating that the events of luteolysis are coordinated to induce cell death without a massive inflammatory response, rather than simply being a cessation of all cellular functions. In the progression from day 12 to day 16 of pregnancy, 52 proteins were upregulated and 14 were downregulated, suggesting that the presence of an embryo suppressed the changes in protein abundance that were apparent when the CL regressed. Upregulated proteins during pregnancy are involved in signal transduction, protein synthesis, electron transfer, steroidogenesis, and cytokine signaling (Arianmanesh *et al*., 2011).

Chung *et al*. (2012) compared the CL of day 90 of pregnancy to midcycle (days 6-13) CL from nonbred cattle. Analysis of the 2D gels revealed differences in protein abundance represented by 32 spots, and from these, 23 proteins were identified, of which 6 were more abundant and 17 were less abundant in CL of pregnancy. Differences in proportions of up- and down- regulated proteins in these two studies are likely due to the different stages from which the CL of pregnancy were collected.

**Figure 4:**
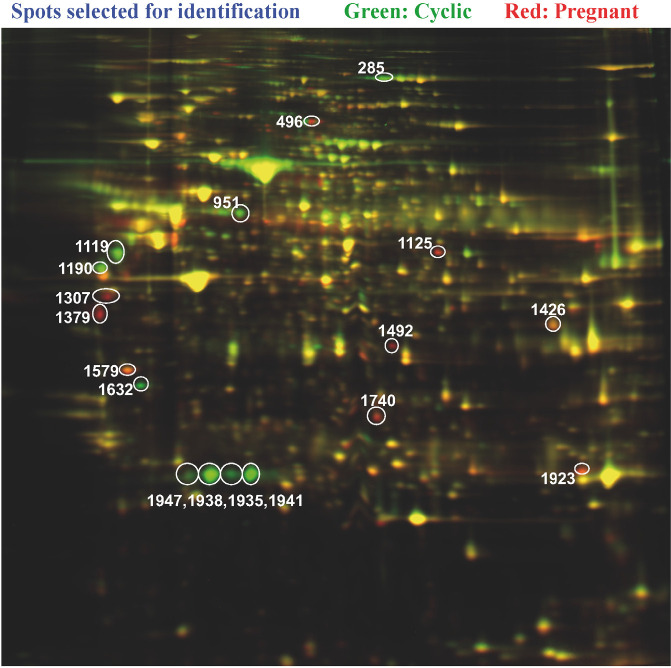
Representative 2D gel of proteins from CL collected on day 18 of the estrous cycle (green dye) and day 18 of pregnancy (red dye). Yellow indicates proteins that were of similar abundance in both treatment groups.

We have also used this procedure to compare bovine CL collected on day 18 of the cycle or pregnancy. The number of differentially expressed proteins was undetermined, but 18 spots that were clearly different in CL from cyclic or pregnant cattle (Fig. 4) were sequenced. Identified differentially abundant proteins included vimentin, adrenodoxin, 3- hydroxymethylglutaryl-CoA synthase, apolipoprotein A1, annexin and glutathione S-transferase. There was considerable similarity in identified proteins between this study and the previous two. Six proteins were differentially abundant in at least two of these three proteomics studies (Arianmanesh *et al*., 2011; Chung *et al*., 2012; Pate *et al*., 2018; Penn State University, Center for Reproductive Biology and Health, University Park, PA USA; unpublished data). Commonly identified proteins among at least two of these three studies are listed in Table 3. Gene family was assigned by Ingenuity Pathway Analysis (IPA; Qiagen). The Diseases and Functions feature of IPA was used to identify functions associated with the commonly differentially abundant proteins. All significant (P< 0.005) functions involving all six differentially abundant proteins and relevant functions involving four or five differentially abundant proteins are shown in Table 4. This analysis demonstrates that proteins that are modulated in the CL during early pregnancy are likely involved in regulating apoptosis and cell survival and maintaining steroidogenesis. Interestingly, all six common proteins were predicted to be involved in synthesis of lipid, which can be addressed in metabolomic studies.

**Table 3:** Proteins identified as differentially abundant during the estrous cycle and pregnancy in at least two proteomics studies.

Protein	Gene symbol	Gene family
Vimentin	VIM	other
Apolipoprotein A1	APOA1	transporter
Annexin (5 or A1)	ANXA1, ANXA5	enzyme
Adrenodoxin reductase	FDXR	enzyme
Glutathione S-transferase	GSTA1	enzyme
Superoxide dismutase	SOD1	enzyme

**Table 4:** Functional analysis of proteins differentially abundant in the estrous cycle and pregnancy in at least two proteomics studies

Functions involving all 6 common proteins	Apoptosis, necrosis, synthesis of lipid
Relevant functions involving 4 or 5 common proteins	Fatty acid metabolism, migration of cells, synthesis of reactive oxygen species, synthesis of steroid hormone, vasculogenesis

## Metabolomic studies of the corpus luteum

Metabolomics is a broad term that can refer to measurement of metabolites of any biochemical processes, including amino acids, sugars, and lipids. Changes in specific lipid metabolites, such as prostaglandins, phospholipids, and steroidal molecules, as the CL progresses from development through luteolysis have been achieved using various targeted approaches, such as chromatography and radioimmunoassay. However, no comprehensive metabolomic study of the CL has been reported. We have recently completed a comprehensive analysis of lipid metabolite concentrations in the CL during the estrous cycle, luteolysis and maternal recognition of pregnancy (Hughes *et al*., 2018; Penn State University, Center for Reproductive Biology and Health, University Park, PA USA; unpublished data). Among 79 lipids measured, there were 24 lipids that differed in abundance during the estrous cycle, all being less abundant on day 4 than on day 11, with nine remaining high on day 18 of the cycle. During a 24-h time-course of luteolysis, 35 lipids changed, and as might be expected, represented arachidonic acid metabolism and prostaglandin signaling. In the early period of maternal recognition of pregnancy, only subtle changes in mRNA, miRNA and proteins are detectable in the CL, and this was reflected in the metabolic profile of the bovine CL on day 18.

**Figure 5:**
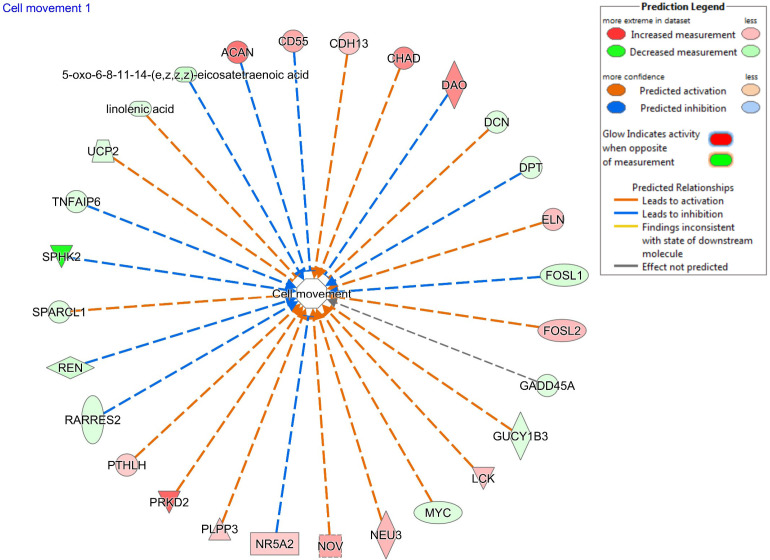
The network from the Diseases and Functions feature of Ingenuity Pathway Analysis (Qiagen) with the greatest total number of molecules, including both differentially abundant genes and lipids from early pregnancy. Red indicates a molecule greater in pregnancy, while green indicates lesser in pregnancy. An orange line indicates activation of cell movement by a molecule, while a blue line indicates inhibition of cell movement.

While there are many programs available for functional and pathway analysis of transcriptomics and proteomics data, fewer such programs are available for analysis of metabolomic data and integration of metabolomic and transcriptomic data. Using the data integration feature in the program MetaboAnalyst (Xia and Wishart, 2016), pathways including sphingolipid (ceramide) metabolism, propanoate metabolism, and pyruvate metabolism were indicated as differentially regulated in the CL of pregnancy. However, close examination of these results revealed that these pathways were modulated by either genes or lipids, but not both, indicating that this program resulted in a listing of potential metabolic pathway modulation, without lipid-metabolite integration. In IPA, the Network and Diseases and Functions features were much more useful in demonstrating functions that may be regulated by combinations of differentially abundant genes and lipids. The top networks containing both differentially abundant genes and lipids were Lipid metabolism, molecular transport, and small molecule biochemistry and DNA replication, recombination and repair, cell death and survival, cellular function and maintenance. Further, differentially abundant mRNA and lipids were expected to be involved in cell movement and migration; among significant diseases or functions annotations, these pathways included the greatest total number of molecules, including both mRNA and lipids (Fig. 5). Other pathways were related to cell interaction and to immune cell differentiation.

## Conclusion

**Figure 6:**
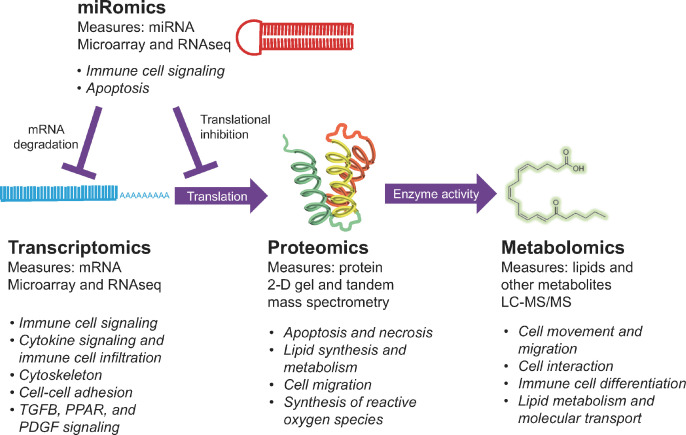
miRomics, transcriptomics, proteomics, and metabolomics have all been used to study the CL of pregnancy. Each technology is shown, with functions modulated in early pregnancy that have been revealed by each technology in italics. miRNA may lead to mRNA degradation or to translational inhibition. mRNA are translated into proteins and proteins mediate the production of lipids and other metabolites that may have key signaling functions.

Although large-scale molecular profiling studies have largely supported existing hypotheses about luteal function, they have also allowed identification of novel signaling pathways that could be targeted to support luteal function. In particular, these studies have contributed to our understanding of the CL of pregnancy, which, because of the subtlety of the changes that occur in these CL, have been difficult to study using targeted approaches in the past. Figure 6 shows a summary of what large-scale molecular profiling and pathway analysis has revealed about functions that are modulated in the CL during early pregnancy. Notably, each of these technologies, except proteomics, have indicated that luteal immune cell function may be modulated during early pregnancy (with proteomics suggesting cell migration, which is likely migration of immune cells), a compelling finding in light of the growing body of evidence that immune cells are intricately involved in regulation of luteal functions.

Application of large-scale molecular profiling to study the corpus luteum is still in a nascent stage, and laboratory experiments to confirm functions suggested by these kinds of studies are necessary to elucidate specific regulation of luteal function. However, improvements in statistical analysis techniques, as well as advances in the profiling technologies themselves, to improve accuracy and precision with which miRNA, mRNA, proteins, and metabolites can be measured, will continue to drive these kinds of studies forward and allow generation of high-quality, high-resolution data, to more completely understand luteal function.
